# Long-Lasting Unruptured Large Intracranial Aneurysm in Bilateral Moyamoya Disease: Case Report

**DOI:** 10.1155/2022/2635724

**Published:** 2022-09-19

**Authors:** Eunyoung Chae, Soomin Jeong, Chan-Hyuk Lee

**Affiliations:** ^1^Medical School, Jeonbuk National University, Jeonju, Republic of Korea; ^2^Department of Neurology, Asan Medical Center, Seoul, Republic of Korea

## Abstract

**Introduction:**

The rupture risk of intracranial aneurysms in patients with moyamoya disease is higher than that in the general population. We report a confirmed case of moyamoya disease with bilateral middle cerebral artery (MCA) occlusion with a large and long-lasting aneurysm.

**Case:**

A 71-year-old woman visited the clinic with a large intracranial aneurysm. The patient was diagnosed with an ischemic stroke 2 months ago. She exhibited weakness in the left upper and lower extremities and dysarthria and was taking aspirin. The brain magnetic resonance imaging showed complete occlusion in the bilateral MCA proximal (M1) and a large 11 × 11 mm nonruptured cerebral aneurysm in the A3 segment of the left anterior cerebral artery. On transfemoral cerebral angiography, the patient was diagnosed with Suzuki grade VI moyamoya disease with bilateral MCA occlusion. After 7 years, the cerebral aneurysm size further increased, but it remained unruptured.

**Conclusions:**

Here, the patient had moyamoya disease with a large aneurysm, but aneurysmal rupture did not occur even after 7 years. Our case report might help in understanding the mechanisms of cerebral aneurysm occurrence and rupture in moyamoya patients.

## 1. Introduction

Moyamoya disease is a chronic cerebrovascular disease involving slowly progressing cerebral artery stenosis, including the Willis' circle, without clear pathophysiology. Although its incidence is high in Asian countries, it has been reported worldwide. The incidence of intracranial aneurysms in moyamoya patients (3.4–14.8%) is higher than that in the general population (1–3%). Additionally, the rupture risk of intracranial aneurysms is high in moyamoya patients [1]. Here, we report a patient diagnosed with moyamoya disease with bilateral middle cerebral artery (MCA) occlusion and a large aneurysm in the left A3 segment, which has not ruptured for >7 years.

## 2. Case Presentation

**Figure 1:**
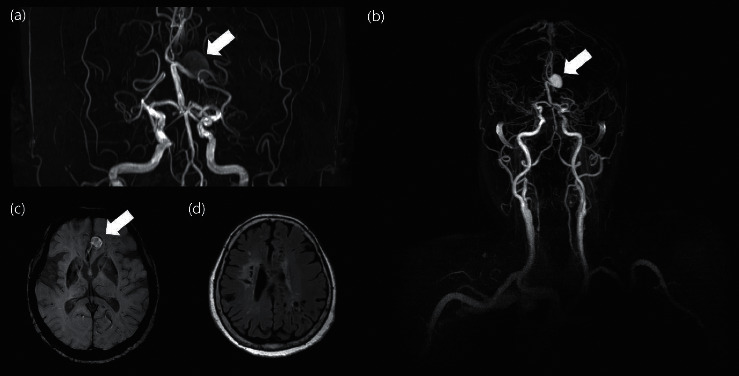
The brain MR images in moyamoya disease patients with a large intracranial aneurysm. (a) Brain TOF-MRA presenting a bilateral middle cerebral artery occlusion with a large aneurysm (arrow). (b) Brain MRA with gadolinium enhancement in the same patient with a large aneurysm (arrow). (c) SWI image presenting with large intracranial aneurysms (arrow). (d) FLAIR image showing multiple old ischemic lesions in the parenchyma.

A 71-year-old woman presented with an intracranial aneurysm. The patient was earlier diagnosed with acute ischemic stroke in the right periventricular white matter and centrum semiovale at another hospital 2 months ago after exhibiting weakness in the left upper and lower extremities and was prescribed aspirin. On admission, mild dysarthria and left upper and lower extremity muscle weakness (Medical Research Council Grade IV) were observed. The brain magnetic resonance imaging (MRI) showed complete occlusion in the bilateral proximal MCA (M1) and a large 11 × 11 mm nonruptured cerebral aneurysm in the A3 segment of the left anterior cerebral artery (ACA) (Figure 1). Perfusion delay was observed in both MCA regions on the brain perfusion MRI. On transfemoral cerebral angiography, the patient was diagnosed with Suzuki grade VI moyamoya disease with bilateral MCA occlusion. After 7 years, a brain MRI was performed for the observation of the cerebral aneurysm. The aneurysm increased to 17 × 13 mm in size, but it remained unruptured. The patient provided informed consent, and the study conforms to the provisions of the Declaration of Helsinki (as revised in Tokyo 2004).

## 3. Discussion

Long-term hemodynamic changes due to major cerebrovascular occlusion may lead to a high frequency of intracranial aneurysms in moyamoya patients. High shear stress not only affects the early development of aneurysms in moyamoya patients, but it also increases the levels of nitric oxide synthase, matrix metalloproteinase-2, and -9 in the aneurysmal tissue, impairing endothelial cell homeostasis. [2].

The high incidence of intracranial aneurysm in moyamoya patients may be associated with the pathological similarities between the two diseases (intracranial aneurysm and moyamoya disease). Decreased mesothelial thickness and structural defects are pathological findings in the intracranial aneurysm. In moyamoya disease, hyperproliferative vascular smooth muscle cells invade the endothelium, destroying the vascular endothelium and narrowing the lumen. The vascular mesothelium becomes fibrous and weak. Therefore, both aneurysms and moyamoya disease cause destruction of the internal elastic lamina and weaken the vascular mesothelium. Recently, derangement of the peri-endothelial matrix in moyamoya disease also increased the fragility by wall shear stress. [3].

Researchers at Nara University conducted a retrospective analysis of 111 moyamoya patients with cerebral aneurysms to study the location of the accompanying cerebral aneurysms. Of the 131 aneurysms identified in the patients, 56% were located in the circle of Willis, 18% in the basal ganglia, and 26% in the collateral vessel. In the circle of Willis, the posterior part was most common (33%), followed by the anterior part (23%). In a similar study on 2,230 moyamoya patients at Zhengzhou University Hospital, 209 cerebral aneurysms were found in 182 (8.2%) patients. Among them, 53 cases were present in the internal carotid artery, 47 cases in the anterior communicating artery or ACA, and 41 cases in the vertebrobasilar or posterior cerebral artery. Furthermore, aneurysm size >5 mm, irregular shape, and occurrence at the basilar tip or moyamoya vessels were risk factors for rupture in moyamoya patients with cerebral aneurysms.

According to the PHASES score for estimating the rupture risk of cerebral aneurysms, our patient was classified in the high-risk group for rupture (PHASES score of 14) [4]. However, the aneurysm in our patient has not ruptured for many years. We suggest two explanations for this condition. The first explanation is the aneurysmal location. Generally, arterial bifurcation aneurysms have a higher rupture risk due to hemodynamic effects [5]. However, our patient had an aneurysm slightly distal to the single A2 bifurcation. Therefore, the shear stress might have been reduced to some extent due to the turbulent flow in the branching area. In the second explanation, a fairly wide preaneurysmal artery diameter (4.3 mm) might have contributed to lowering the shear stress. A large diameter leads to an increase in the cross-sectional area, which contributes to lowering the shear stress. [6].

Aneurysms of a small diameter could be regressed by surgical procedures. Researchers in Germany investigated the microaneurysms of moyamoya vessels by using MRI. They found that one case of a microaneurysm naturally improved after bypass surgery [7]. Reduced hemodynamic stress might have contributed to the alleviation of the aneurysm.

Here, the patient with moyamoya disease did not experience an aneurysm rupture for a long period despite having a large aneurysm. Previous case studies suggest that various factors are involved in aneurysm rupture in moyamoya patients. This study will help in understanding the mechanisms of cerebral aneurysm occurrence and rupture in moyamoya patients.

## Data Availability

No data were used to support this study.

## Abbreviations

MR: Magnetic resonance
TOF: Time of flight
SWI: Susceptibility weighted imaging
FLAIR: Fluid-attenuated inversion recovery.
